# Inverted Cyclops Lesion of the Knee Associated with a Supracondylar Femoral Nail: A Case Report

**DOI:** 10.7759/cureus.5902

**Published:** 2019-10-14

**Authors:** Srinivas B Kambhampati, Saseendar Shanmugasundaram

**Affiliations:** 1 Orthopaedics, Sri Dhaatri Orthopaedic, Maternity and Gynaecology Center, Vijayawada, IND; 2 Orthopaedics, Apollo Hospital, Muscat, OMN

**Keywords:** knee stiffness, rehabilitation, gait, cyclops lesion, inverted cyclops lesion, knee, supracondylar nailing

## Abstract

While cyclops lesion, a fibrous nodule on the tibial side of the knee joint, is a well-known condition complicating anterior cruciate ligament, inverted cyclops lesion, a fibrous nodule on the femoral side of the knee, is a relatively less known condition. We report a case of inverted cyclops in a patient who presented with chronic knee stiffness eight years after supracondylar nailing of a femoral shaft fracture. There are only four reported cases of inverted cyclops in literature. Literature has been reviewed and the importance of not missing such a lesion is discussed.

## Introduction

Cyclops lesion is a distinct entity, known to occur after anterior cruciate ligament (ACL) reconstruction, and commonly leads to extension block of the knee. The lesion is a fibrous nodule of granulation tissue that is similar to a healing scar, arising from the tibia, with occasional cartilaginous or bony tissue within it [1].

While cyclops lesion is reported to have an incidence of up to 10% in the literature, inverted cyclops lesion is a rare condition that has been reported only four times so far, including a report by one of the authors of the current report [1-4]. Cyclops and inverted cyclops is typically associated with an ACL injury and reconstruction. Although supracondylar nailing for fracture shaft of the femur is being done for a few years, there has been no report of such a lesion in the literature. We report one case of inverted cyclops lesion and an impinging intra-articular heterotopic bone in the knee seen during arthroscopy eight years after a supracondylar nail for a femoral shaft fracture, complicated by post-operative infection.

## Case presentation

A 40-year-old man, a temple priest by profession, presented with stiffness in his right knee for eight years. He had a motor vehicle accident (MVA) in 2011 when the auto-rickshaw in which he was traveling toppled and he sustained a fracture of both bones of his right forearm, fracture of the shaft of the right femur as well as basicervical fracture of the neck of right femur. He had undergone plating of both bones of the forearm, dynamic hip screw (DHS) for the neck of femur fracture and supracondylar intramedullary nailing for fracture shaft of the femur. The femoral neck and forearm fractures went on to heal uneventfully. However, he developed an infection of the femoral shaft fixation with a persistent discharging sinus from the thigh for six months after the surgery. The fracture went into non-union but the sinus healed and hence he had another surgery for bone grafting. The femoral shaft fracture united six months after bone grafting and one year after MVA. At this visit, he presented with stiffness in the knee and a short right lower limb.

On examination, he had a puckered scar on the lateral aspect of his thigh indicating a healed infected sinus, in addition to two surgical scars - one proximal for the DHS and the other distal for the interlocking screw proximally and bone graft surgery. The knee was stiff with flexion of 30 degrees from flexion deformity of 10 degrees, with a very firm endpoint. The right knee examination revealed laxity of the posterior cruciate ligament. Anterior cruciate ligament and both collaterals appeared intact. There was no distal neurovascular deficit. The right thigh was short by two centimeters.

**Figure 1:**
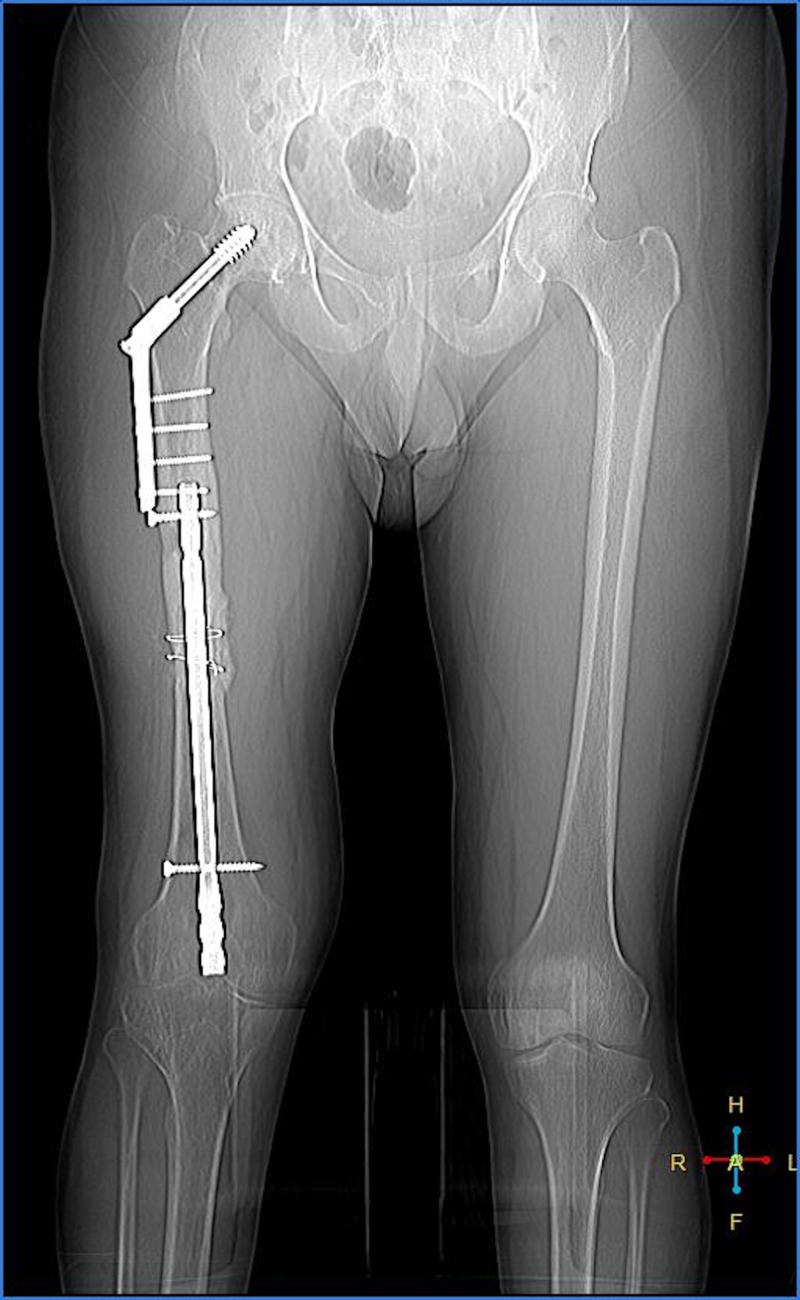
Computed tomography scanogram of the right lower limb with implants in situ

A computed tomography scanogram of the right thigh and knee showed a united femoral shaft fracture with no evidence of loosening of the implant or osteomyelitis. The DHS was intact with no evidence of loosening (Figure 1).

**Figure 2:**
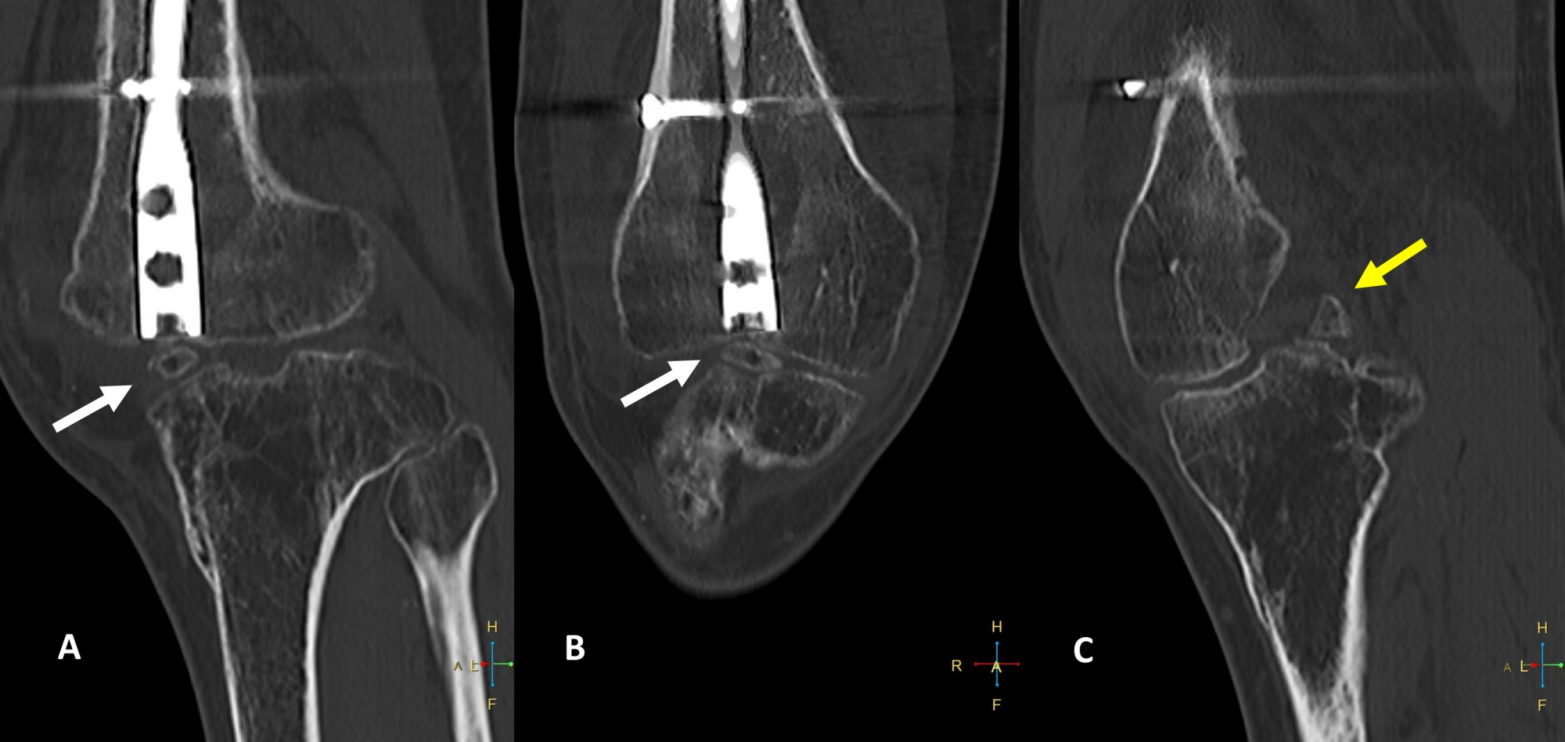
Two-dimensional computed tomography images of the knee A: coronal image showing heterotopic bone inferior to the lower end of the nail. B: sagittal section showing the distal nail and heterotopic bone in relation to the end of the nail. C: shows nonunion of avulsion fracture of PCL. White arrow - heterotopic bone; yellow arrow - PCL avulsion; PCL - posterior cruciate ligament

Two-dimensional computed tomography scan of the femur revealed heterotopic bone formation in the knee just posterior to the infrapatellar fat pad region at the tip of the nail (Figure 2).

**Figure 3:**
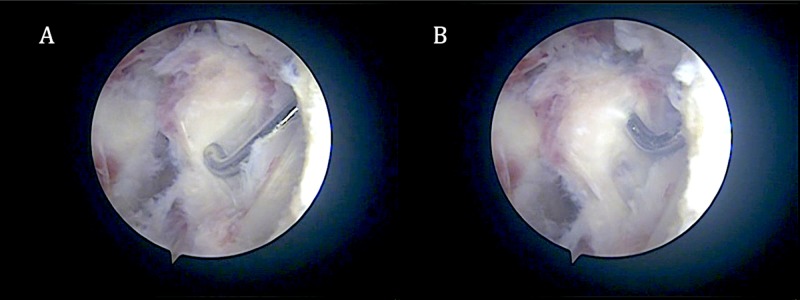
Arthroscopic picture of the infrapatellar piece of heterotopic bone during (A) and after (B) mobilization before excision Note the amount of fibrous tissue around the bone.

Arthroscopy of the knee revealed heterotopic bone formation inferior to the lower end of the nail, adherent to the tibia (Figure 3).

**Figure 4:**
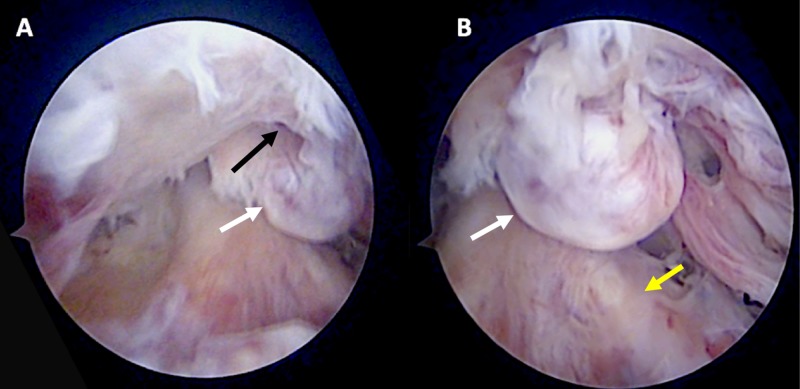
Arthroscopic images of the inverted cyclops lesion in relation to the femoral condyles (A) and in relation to the anterior cruciate ligament (B) It must be noted that there is associated fibrosis from the previous intra-articular approach to place the nail. White arrow - heterotopic bone; black arrow - femoral notch; yellow arrow - anterior cruciate ligament

The fragment was impinging on the anterior aspect of the femoral notch, where a soft-tissue nodule arising from the femur had formed (inverted cyclops) (Figure 4).

The heterotopic bone, as well as the inverted cyclops nodule, were excised. Arthroscopic release of fibrous adhesions followed by gentle manipulation of the knee was done. Supracondylar nail was removed after the arthroscopy and manipulation. A knee range of motion of 0-120 degrees was obtained. Extensive post-operative rehabilitation was given to improve the range of motion. At the last follow-up at three months, he had no flexion deformity and further flexion was 120 degrees. He did not have instability of the knee with regular daily activities and was not keen on proceeding with the reconstruction of the posterior cruciate ligament.

## Discussion

We did a literature search in Pubmed, Medline, and Scopus for "inverted cyclops lesion". Bibliography of the articles was further searched for relevant articles. In all, we found four reports of inverted cyclops lesions. Inverted cyclops lesion has been reported following distal femoral physeal fracture, in association with ACL reconstruction with bone-patella tendon-bone graft or hamstrings graft [4-7]. All patients except one had an extension deficit. However, inverted cyclops lesion associated with a kissing intra-articular infrapatellar bone fragment following supracondylar nailing of the femur has not been reported so far.

The origin of inverted cyclops has been varied. While Rubin et al. reported the nodule to be arising from the femoral tunnel, Kambhampati et al. reported no communication between the nodule and the femoral tunnel [4, 6]. The origin of the cyclops was confirmed both by magnetic resonance imaging (MRI) and by arthroscopy in these reports.

It has been postulated from the previous case reports that the origin of the cyclops arises from the drilling debris of the tunnels. For the regular cyclops lesion, the debris comes from the tibial tunnel and for the inverted cyclops lesion, the debris is thought to be arising from the femoral tunnel.

The origin of the lesion in the present case can be two-fold: 1) fibrous tissue extending from the femoral nail entry, and 2) reactive fibrous tissue secondary to impingement from heterotopic bone lying on the intraarticular portion of anterior tibia. The lesion does not appear to be related to the musculoskeletal infection, as there was no history, clinical finding or radiological evidence of sepsis of the knee joint. None of the previous reports were associated with musculoskeletal infection.

We advise a high index of suspicion for an inverted cyclops lesion in patients who present with a fixed flexion deformity following supracondylar nailing for femoral shaft fracture. These may only be diagnosed by arthroscopy of the knee as magnetic resonance imaging is unlikely to be helpful to detect the lesion due to metal artifacts from the presence of the nail. Musculoskeletal ultrasound of the knee could help in making a diagnosis, though it was not used in the present scenario due to a lack of suspicion. Further studies are required to find out the true incidence of this lesion after supracondylar nailing of the femur. An MRI is advisable for all patients with persistent postoperative flexion deformity of the knee that does not respond to physiotherapy. If present, these patients are unlikely to improve significantly unless the lesion is excised.

Various recommendations have been made to reduce the incidence of cyclops lesion in the literature. These measures include minimally invasive surgery, proper tunnel position to avoid impingement, creating less debris and clearing all the debris after tunnel placement, early postoperative mobilization and notchplasty [8, 9].

## Conclusions

We advise clearing of bone debris generated from reaming for the supracondylar nail and placing a nail cap to reduce the load of further bone marrow cells and debris discharge into the joint to prevent the inverted cyclops lesion in these cases. Avoiding the prominence of the implant in the joint is also imperative.
